# Acetic Acid Causes Endoplasmic Reticulum Stress and Induces the Unfolded Protein Response in *Saccharomyces cerevisiae*

**DOI:** 10.3389/fmicb.2017.01192

**Published:** 2017-06-28

**Authors:** Nozomi Kawazoe, Yukio Kimata, Shingo Izawa

**Affiliations:** ^1^Laboratory of Microbial Technology, Graduate School of Science and Technology, Kyoto Institute of TechnologyKyoto, Japan; ^2^Graduate School of Biological Sciences, Nara Institute of Science and TechnologyNara, Japan

**Keywords:** acetic acid, lactic acid, ER stress, unfolded protein response, Ire1p, Hac1p, BiP, *Saccharomyces cerevisiae*

## Abstract

Since acetic acid inhibits the growth and fermentation ability of *Saccharomyces cerevisiae*, it is one of the practical hindrances to the efficient production of bioethanol from a lignocellulosic biomass. Although extensive information is available on yeast response to acetic acid stress, the involvement of endoplasmic reticulum (ER) and unfolded protein response (UPR) has not been addressed. We herein demonstrated that acetic acid causes ER stress and induces the UPR. The accumulation of misfolded proteins in the ER and activation of Ire1p and Hac1p, an ER-stress sensor and ER stress-responsive transcription factor, respectively, were induced by a treatment with acetic acid stress (>0.2% v/v). Other monocarboxylic acids such as propionic acid and sorbic acid, but not lactic acid, also induced the UPR. Additionally, *ire1*Δ and *hac1*Δ cells were more sensitive to acetic acid than wild-type cells, indicating that activation of the Ire1p-Hac1p pathway is required for maximum tolerance to acetic acid. Furthermore, the combination of mild acetic acid stress (0.1% acetic acid) and mild ethanol stress (5% ethanol) induced the UPR, whereas neither mild ethanol stress nor mild acetic acid stress individually activated Ire1p, suggesting that ER stress is easily induced in yeast cells during the fermentation process of lignocellulosic hydrolysates. It was possible to avoid the induction of ER stress caused by acetic acid and the combined stress by adjusting extracellular pH.

## Introduction

Acetic acid is a potent inhibitor of the growth and fermentation ability of the budding yeast *Saccharomyces cerevisiae* (Arneborg et al., 1995; Phowchinda et al., 1995; Pampulha and Loureiro-Dias, 2000; Narendranath et al., 2001; Vilela-Moura et al., 2011). Since hemicellulose and lignin are highly acetylated, acetic acid is released during the pretreatment of the lignocellulosic biomass prior to bioethanol production and remains in hydrolysates (Palmqvist and Hahn-Hägerdal, 2000; van Maris et al., 2006; Vilela-Moura et al., 2011; Koppram et al., 2014). Although the concentration of acetic acid in the lignocellulosic hydrolysates varies widely according to the types of treatment methods and biomass used, a wide range of acetic acid concentrations (0*–*178 mM) was reported in previous studies (Klinke et al., 2004; Nakata et al., 2006; Almeida et al., 2007). Additionally, acetic acid is easily produced in open fermenters due to contamination by lactic acid bacteria and acetic acid bacteria. Since the toxicity of acetic acid is one of the serious practical hindrances to reducing the production cost of bioethanol from the lignocellulosic biomass (Koppram et al., 2014), improving the acetic acid tolerance of yeast is strongly desired.

In order to improve yeast tolerance to acetic acid, better understandings of the physiological effects of acetic acid and cellular responses to acetic acid stress are essential. In low pH conditions below the pKa value (<4.76), the undissociated form of acetic acid can enter the yeast cells by simple diffusion or through the aquaglyceroporin Fps1p (Guldfeldt and Arneborg, 1998; Mollapour and Piper, 2007). Once in the near-neutral cytoplasm, acetic acid dissociates into protons (H^+^) and acetate anions (CH_3_COO^-^), following the acidification of the cell interior (Guldfeldt and Arneborg, 1998; Arneborg et al., 2000). Due to their electric charges, protons and acetate anions cannot permeate the plasma membrane by simple diffusion. In order to maintain the pH in the cytoplasm, the accumulated protons can be excluded from the cytoplasm by the plasma membrane H^+^-ATPase (Pma1) and Vacuolar H^+^-ATPase (Carmelo et al., 1997; Martinez-Munoz and Kane, 2008). Acetate anions can be extruded from the cells by ATP-binding cassette (ABC) transporters such as Pdr12p, and therefore the null mutant of *PDR12* shows hypersensitivity to acetic acid (Holyoak et al., 1999). Intracellular acetate anions can also be metabolized through the conversion to acetyl-CoA (Vilela-Moura et al., 2008; Lee et al., 2011). Although *S. cerevisiae* has monocarboxylate transporters, Ady2p and Jen1p, which can transport acetate anions into the cells, their expressions are strictly repressed in the presence of glucose (Casal et al., 1996; Giannattasio et al., 2013). Therefore, it is considered that exponentially growing cells in the presence of glucose do not take in extracellular acetate anions.

Reported that very high concentrations of acetic acid induced programmed cell death in yeast cells (Ludovico et al., 2001; Giannattasio et al., 2013). Several genome-wide analyses clarified the key roles of Haa1p, a weak-acid responsive transcription factor, in tolerance to acetic acid (Fernandes et al., 2005; Mira et al., 2010b, 2011; Haitani et al., 2012). Since the transcription of 80% of acetic-acid-responsive genes is activated in a Haa1p-dependent manner (Mira et al., 2010b, 2011), the overexpression of the *HAA1* gene enhances tolerance to acetic acid (Tanaka et al., 2012; Inaba et al., 2013). On the other hand, Lindahl et al. (2016) recently reported that sphingolipid levels in the membrane influence yeast tolerance to acetic acid. We also demonstrated that Mrh1p and Yro2p are involved in tolerance to acetic acid stress (Takabatake et al., 2015). Furthermore, Fernández-Niño et al. (2015) showed that cytosolic pH prior to treatment with acetic acid affected acetic acid tolerance.

Although knowledge on the key factors involved in the acetic acid tolerance of yeast has been increasing (Mira et al., 2010b,a; Sousa et al., 2012; Giannattasio et al., 2013; Geng et al., 2017), the involvement of endoplasmic reticulum (ER) and unfolded protein response (UPR) has not been addressed. Since vinegar is known to cause protein denaturation via examples of poached eggs and pickled herring, we here examined whether acetic acid causes the UPR (Mori, 2009; Gardner et al., 2013). In eukaryotic cells, newly synthesized secretory and transmembrane proteins are folded and assessed for their quality in the ER (Brodsky and Skach, 2011), and the accumulation of misfolded proteins in the ER (ER stress) evokes the UPR (Mori, 2009; Pincus et al., 2010; Gardner et al., 2013; Wu et al., 2014). In *S. cerevisiae*, the UPR is triggered by the ER-located transmembrane RNase Ire1p through splicing of the *HAC1* mRNA. Ire1p senses unfolded proteins in the ER and is activated via the self-association and formation of oligomers (Kimata et al., 2007). It has been clarified that highly oligomerized Ire1p-GFP exhibits punctate intracellular localization, although Ire1p-GFP diffuses throughout the ER under non-stressed conditions (Kimata et al., 2007; Aragón et al., 2009; Promlek et al., 2011). The active form of Ire1p splices the *HAC1*-gene transcript (*HAC1*^u^) to yield translatable mRNA (*HAC1*^i^), which may be translated into the transcription factor Hac1p (Chapman and Walter, 1997; Kawahara et al., 1997). Hac1p activates the transcription of a number of UPR-related genes including *KAR2*, which encodes the major ER chaperone BiP (Mori et al., 1992). BiP forms aggregates incorporating misfolded proteins in the ER (Kimata et al., 2003; Promlek et al., 2011; Nguyen et al., 2013; Miyagawa et al., 2014). Therefore, it is possible to monitor yeast UPR by analyzing the levels of unfolded protein aggregation in the ER, the oligomerization of Ire1p, splicing of *HAC1* mRNA, and *KAR2* expression.

In the present study, we found that acetic acid led to elevated levels of unfolded protein aggregation in the ER and caused the activation of the Ire1p-Hac1p pathway, clearly indicating that acetic acid induced the UPR in yeast. We also examined whether the combination of mild acetic acid stress and mild ethanol stress causes ER stress. Although neither stress alone induced the oligomerization of Ire1p, their combination did. These results clearly indicate that one of the adverse effects of acetic acid is the induction of ER stress. Our results suggest that ER stress is easily induced in yeast cells in fermenting lignocellulosic hydrolysates and that strengthening the capacity to cope with ER stress may contribute to improving the acetic acid tolerance of yeast cells.

## Materials and Methods

### Strains and Medium

The *S. cerevisiae* strain KMY1015 (*MATa leu2-3, 112 ura3-52 his3-200*Δ *trp1-*Δ*901lys2-801 ire1*Δ*::TRP1* carrying the *IRE1-GFP* plasmid) was used in the present study (Ishiwata-Kimata et al., 2013). BY4742 (*MATα his3*Δ*1 leu2*Δ*0 lys2*Δ*0 ura3*Δ*0*) and its isogenic *ire1*Δ and *hac1*Δ null mutants (Open Biosystems Inc., Huntsville, AL, United States) were also used for the stress tolerance assay. BY4742, *ire1*Δ, and *hac1*Δ cells were cultured in 50 ml of SD medium (2% glucose, 0.67% yeast nitrogen base w/o amino acids, 20 mg/L uracil, 30 mg/L L-lysine HCl, 100 mg/L L-leucine, and 20 mg/L L-histidine HCl, pH 5.30) at 28°C with reciprocal shaking (120 rpm) in Erlenmeyer flasks (200 ml). As described in Ishiwata-Kimata et al. (2013), the *IRE1-GFP* plasmid carried in KMY1015 is based on the *HIS3* single-copy vector pRS313 (Sikorski and Hieter, 1989). In order to prevent the plasmid loss, KMY1015 cells were cultured in SD medium without L-histidine. Exponentially growing cells were harvested at OD_600_ = 0.5 and exposed to stress. The dissociated form of acetic acid was obtained by using SD-PPB medium (pH 6.80), which was prepared using 100 mM potassium phosphate buffer (PPB, pH 6.80) instead of water. The final pH values after addition of acetic acid and ethanol into the SD-PPB medium were indicated in **Figure 7**. Organic acids [acetic acid, Wako 012-00245, 99.9+%(Ti); lactic acid, Wako 128-00056, 85∼92%(Ti); propionic acid, Wako 169-04723, 98+%(cGC); sorbic acid, Wako 190-03732, 98+%(Ti)] and DTT were obtained from Wako Pure Chemical Industries (Osaka, Japan).

### Stress Tolerance Assay

Cells cultivated in SD medium until OD_600_ = 0.5 were harvested and resuspended in fresh SD medium to obtain an initial OD_600_ value of 0.1. To examine their susceptibility to acetic acid, the cells were treated with 0.3% acetic acid for 5 h at 28°C with reciprocal shaking (120 rpm). In the spot test assay, samples were spotted (10 μl) onto SD agar plates in 10-fold serial dilutions and incubated at 28°C for 2 days. In the survival assay, samples were diluted 500-fold and aliquots (100 μl) were plated onto YPD agar plates. Relative survival rate was calculated as colony-forming units (CFUs).

### Protein Analysis

A BiP aggregation analysis was performed using the method of Promlek et al. (2011). Briefly, total cell lysates were immediately prepared using buffer A (50 mM Tris-HCl, 5 mM EDTA, and 1% Triton X-100, pH 7.9) after the treatment with acetic acid or DTT. Total cell lysates were fractionated by centrifugation at 19,300 × *g* for 10 min, and supernatant fractions and pellet fractions were analyzed by Western blotting using 8% polyacrylamide gels and an anti-BiP antibody (Promlek et al., 2011). The bands of the Western blotting were quantified using ImageJ software^1^. Protein loading abundance was verified and normalized by Ponceau S staining.

### Quantitative Real-Time PCR and *HAC1* mRNA Analysis

The relative mRNA levels of the *KAR2* and *ACT1* genes were assessed by quantitative real-time PCR (qRT-PCR). The method of qRT-PCR was previously described (Yamauchi and Izawa, 2016). The oligonucleotide sequences of the primers used for qRT-PCR were as follows: *KAR2*, 5′-AGACTAAGCGCTGGCAAGCT-3′ and 5′-ACCACGAAAAGGGCGTACAG-3′; *ACT1*, 5′-TTGGATTCCGGTGATGGTGTTACT-3′ and 5′-TGAAGAAGATTGAGCAGCGGTTTG-3′ (Takahashi et al., 2011; Cakir, 2012). The splicing of *HAC1* mRNA was monitored by the methods of Promlek et al. (2011).

### Microscope

A Leica AF6500 fluorescence microscopic system (Leica Microsystems Vertrieb GmbH, Germany) was used in the analysis of Ire1p-GFP. Cells treated with stressors were immediately observed without fixation using the optical magnification of 1000x. In order to obtain the quantitative data, one hundred cells under each condition were examined and experiments were independently repeated three times (300 cells in total were examined).

### Statistical Analysis

**FIGURE 1:**
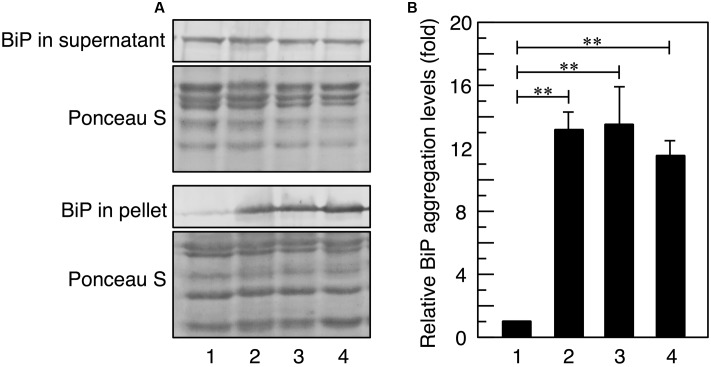
BiP aggregation upon acetic acid stress. Exponentially growing cells were exposed to stress with acetic acid or 10 mM DTT for 3 h and lysed using glass beads in Triton X-100-containing buffer. **(A)** Total cell lysates were fractionated into the supernatant and pellet fractions by centrifugation and analyzed using an anti-BiP antibody. Protein loading abundance was verified and normalized by Ponceaus S staining. **(B)** Protein levels of BiP in the pellet fraction were quantified using ImageJ, and the intensity of the BiP band in cells treated without stress was considered to be 1-fold. Data are shown as the mean ± standard error (*n* = 3). *Lane 1*, w/o stress; *lane 2*, 0.2% acetic acid; *lane 3*, 0.3% acetic acid; *lane 4*, 10 mM DTT. ^∗∗^*P*-value < 0.01.

**FIGURE 2:**
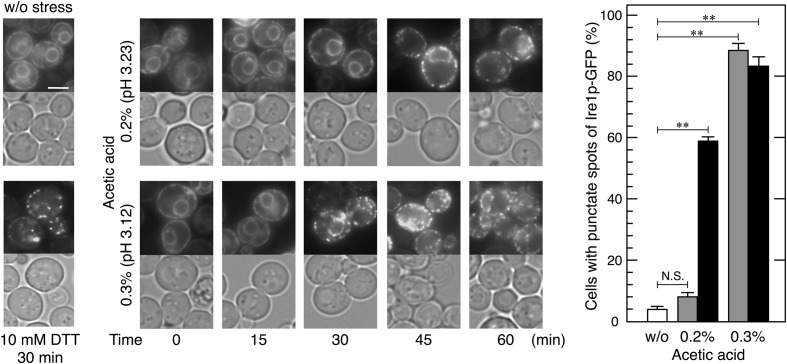
Changes in the localization of Ire1p-GFP upon acetic acid stress. Exponentially growing cells were exposed to the indicated conditions. Ire1p-GFP was immediately observed after the treatment. Representative pictures are shown. **(Upper)**, Ire1p-GFP; **(Bottom)**, bright field. The white bar indicates 3 μm. Quantitative data are shown as means ± standard error. One hundred cells under each condition were examined and experiments were repeated three times (300 cells in total were examined). Cells were treated with acetic acid for 30 min (gray bars) or 60 min (black bars). ^∗∗^*P*-value < 0.01. N.S., no statistically significant difference.

**FIGURE 3:**
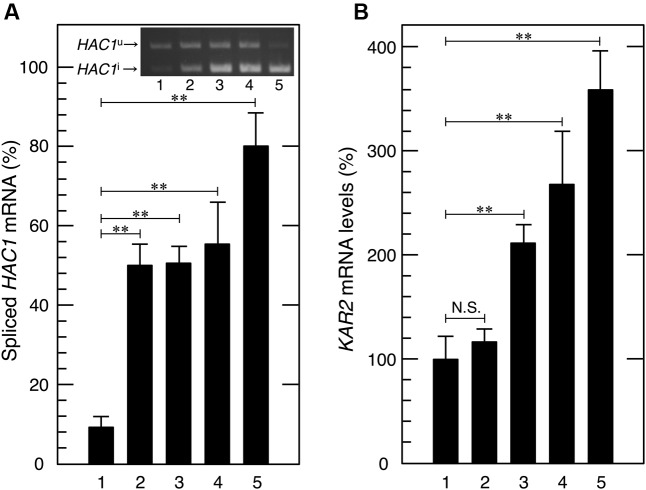
Acetic acid induced the splicing of *HAC1* mRNA and transcriptional activation of *KAR2*, a target gene of Hac1p. Exponentially growing cells were exposed to the indicated stress conditions. **(A)** Total RNA samples from cells treated with acetic acid or DTT were subjected to RT-PCR in order to amplify the *HAC1* products. *HAC1*^u^ and *HAC1*^i^ were fractionated using 2.0% agarose gel electrophoresis. *Lane 1*, w/o stress; *lane 2*, 0.3% acetic acid for 15 min, *lane 3*, 0.3% acetic acid for 30 min; *lane 4*, 0.3% acetic acid for 60 min; *lane 5*, 10 mM DTT for 60 min. **(B)** *KAR2* mRNA levels were analyzed by qRT-PCR. In order to compare expression levels, the mRNA level of *KAR2* was normalized to that of *ACT1* under each condition. The mRNA level in cells without a stress treatment was considered to be 100%. Data are shown as the mean ± standard error (*n* = 3). *Lane 1*, w/o stress; *lane 2*, 0.1% acetic acid for 60 min, *lane 3*, 0.2% acetic acid for 60 min; *lane 4*, 0.3% acetic acid for 60 min; *lane 5*, 10 mM DTT for 60 min. ^∗∗^*P*-value < 0.01. N.S., no statistically significant difference.

**FIGURE 4:**
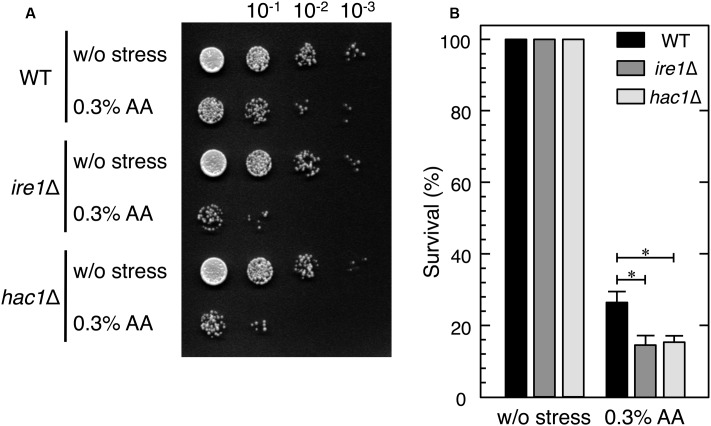
*ire1*Δ and *hac1*Δ cells showed higher susceptibility to acetic acid stress than wild-type cells. Exponentially growing cells (OD_600_ = 0.5) were harvested and resuspended in fresh SD medium to obtain an initial OD_600_ value of 0.1. To examine their susceptibility to acetic acid, the cells were treated with 0.3% acetic acid (AA) for 5 h. **(A)** Samples were diluted in 10^-1^ steps with SD medium, dripped (10 μl) onto SD agar plates, and incubated at 28°C for 2 days. **(B)** Samples were diluted 500-fold and aliquots (100 μl) were plated onto YPD agar plates. Relative survival rates were calculated as colony-forming units (CFUs). The CFUs of cells before the stress treatment was considered to be 100%. Data are shown as the mean ± standard error (*n* = 3). ^∗^*P*-value < 0.05.

**FIGURE 5:**
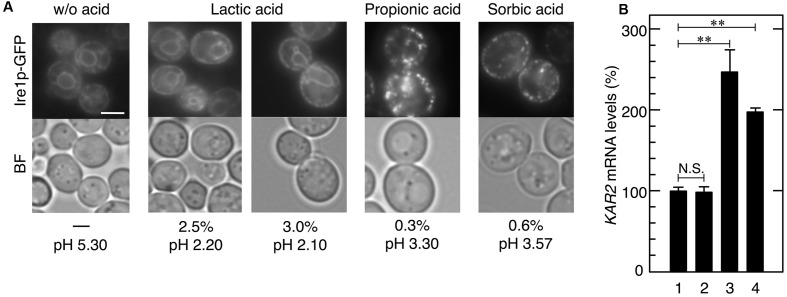
Effects of various carboxylic acids on the induction of UPR. Exponentially growing cells were treated with various carboxylic acids for 30 min. **(A)** Ire1p-GFP was immediately observed after the treatment without fixation. Representative pictures are shown. BF, bright field. The white bar indicates 3 μm. **(B)** The mRNA levels of *KAR2* were analyzed by qRT-PCR. In order to compare expression levels, the mRNA level of *KAR2* was normalized to that of *ACT1* under each condition. The mRNA level in cells without a stress treatment was considered to be 100%. Data are shown as the mean ± standard error (*n* = 3). *Lane 1*, w/o stress; *lane 2*, 3.0% lactic acid, *lane 3*, 0.3% propionic acid; *lane 4*, 0.6% sorbic acid. ^∗∗^*P*-value < 0.01. N.S., no statistically significant difference.

**FIGURE 6:**
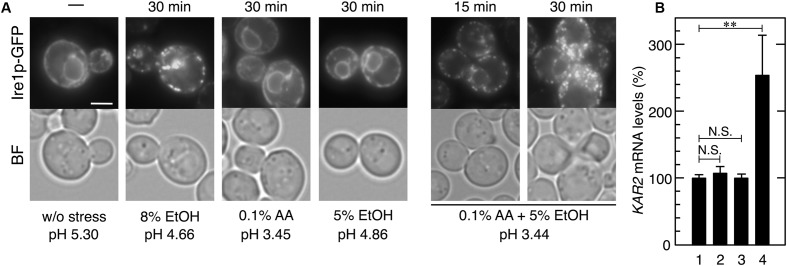
Effects of the combination of mild ethanol stress and mild acetic acid stress on the induction of the UPR. Exponentially growing cells were treated with ethanol (EtOH) and acetic acid (AA). **(A)** Ire1p-GFP was immediately observed after the treatment without fixation. Representative pictures are shown. BF, bright field. The white bar indicates 3 μm. **(B)** The mRNA levels of *KAR2* were analyzed by qRT-PCR. In order to compare expression levels, the mRNA level of *KAR2* was normalized to that of *ACT1* under each condition. The mRNA level in cells without a stress treatment was considered to be 100%. Data are shown as the mean ± standard error (*n* = 3). *Lane 1*, w/o stress; *lane 2*, 0.1% AA for 60 min, *lane 3*, 5% EtOH for 60 min; *lane 4*, 0.1% AA and 5% EtOH for 60 min. ^∗∗^*P*-value < 0.01. N.S., no statistically significant difference.

Unpaired Student’s *t*-tests were performed to analyze the results of **Figures 1**–**6**. Compared results were considered statistically significant when the ^∗^*P*-value < 0.05.

## Results

### Acetic Acid Increased BiP Aggregate Levels in the ER

Since misfolded proteins in the ER often form aggregates with BiP (a major molecular chaperone in the ER), it is possible to estimate the level of ER stress by measuring sedimentable BiP levels (Kimata et al., 2003; Promlek et al., 2011; Nguyen et al., 2013; Miyagawa et al., 2014). Thus, at the beginning of this study, we investigated whether acetic acid actually increased the levels of BiP aggregates. As shown in **Figure 1**, BiP aggregate levels were increased by the treatment with dithiothreitol (DTT), a representative ER stress inducer through interference with disulfide bond formation (Kimata et al., 2003, 2007). Treatments with 0.2 and 0.3% (v/v) acetic acid (35.0 and 52.5 mM, respectively) also increased the levels of BiP aggregates, indicating that acetic acid as well as DTT increased the levels of misfolded proteins in the ER. These results suggest that acetic acid caused ER stress in yeast cells.

### Acetic Acid Activated the Ire1p-Hac1p Pathway

In order to examine whether increases in the levels of BiP aggregates affect Ire1p, we monitored changes in the localization of Ire1p-GFP upon acetic acid stress. Activated Ire1p-GFP is known to be highly self-oligomerized and shows a punctate intracellular localization, while the non-activated form of Ire1p-GFP diffuses throughout the ER (Kimata et al., 2007; Aragón et al., 2009). As previously reported, the Ire1p-GFP signal exhibited double ring-like ER patterns under non-stressed conditions and formed punctate spots after the treatment with DTT (**Figure 2**). In the case of acetic acid stress, the formation of the punctate spots of Ire1p-GFP was significantly induced within 45 min by 0.2% acetic acid and within 30 min by 0.3% acetic acid. These results clearly indicate that acetic acid causes the high-order oligomerization and activation of Ire1p.

We then investigated whether acetic acid causes the splicing of *HAC1* mRNA via RT-PCR amplification and electrophoresis fractionation of the *HAC1* species (Promlek et al., 2011; Nguyen et al., 2013; Miyagawa et al., 2014). We confirmed that the DTT treatment clearly induced the splicing of *HAC1* mRNA (**Figure 3A**). Acetic acid stress (0.3% v/v) also gradually, but significantly converted *HAC1*^u^ to *HAC1*^i^. These results clearly indicate that acetic acid actually activated Ire1p, and this was followed by *HAC1* mRNA splicing.

Since the splicing of *HAC1* mRNA was induced by acetic acid, we examined the activity of Hac1p as an ER stress responsive transcription factor. Under ER stress conditions, the translation product of *HAC1*^i^ has been shown to activate the transcription of its target genes, which contain the UPR element (UPRE) in their promoter regions (Mori et al., 1992). Since the *KAR2* gene is one of the representative target genes of Hac1p and encodes the ER chaperone protein BiP (Rose et al., 1998), we examined the mRNA levels of *KAR2* upon acetic acid stress. After the treatment with acetic acid (0.2 or 0.3%) for 60 min, *KAR2* mRNA levels were more than twofold higher than basal levels (**Figure 3B**), suggesting that acetic acid induced Hac1p protein expression and the transcriptional activation of Hac1p-target genes. Therefore, we concluded that acetic acid caused ER stress and activated the Ire1p-Hac1p pathway.

### *ire1*Δ and *hac1*Δ Cells Showed Higher Susceptibility to Acetic Acid Stress than Wild-Type Cells

We next investigated whether the null mutants of *IRE1* and *HAC1* are hypersensitive to acetic acid stress. The spot test assay for cell viability after the treatment with acetic acid for 5 h demonstrated that *ire1*Δ and *hac1*Δ cells were slightly hypersensitive to acetic acid (**Figure 4A**). The resulting survival rates also demonstrated that *ire1*Δ and *hac1*Δ cells were more susceptible to acetic acid than wild-type cells (**Figure 4B**).

### Effects of Other Organic Acids on the Induction of UPR

We also examined the effects of other carboxylic acids used in food industries, namely lactic acid (p*K*a = 3.86), propionic acid (p*K*a = 4.88), and sorbic acid (p*K*a = 4.76). Although 0.3% propionic acid and 0.6% sorbic acid as well as acetic acid induced the formation of punctate spots of Ire1p-GFP and the transcriptional activation of *KAR2* (**Figure 5**), 0.3–3.0% lactic acid caused no changes in the localization of Ire1p-GFP. Additionally, neither malic acid nor succinic acid induced activation of the Ire1p-Hac1p pathway (data not shown). These results indicate that ER stress induction effects are diverse and depend on the type of organic acid.

### Combination of Mild Acetic Acid Stress and Mild Ethanol Stress Caused the UPR

The combination of mild stresses of different types is known to exert severe adverse effects on cell metabolism such as translation repression (Iwaki et al., 2013; Yamamoto and Izawa, 2013). Additionally, ethanol concentrations gradually increase during the fermentation process in lignocellulosic hydrolysates. We herein examined whether the combination of mild acetic acid stress and mild ethanol stress induces the UPR in yeast. Although 8% (v/v) ethanol induced the formation of punctate spots of Ire1p-GFP (Miyagawa et al., 2014; Navarro-Tapia et al., 2016), neither mild ethanol stress (5% v/v) nor mild acetic acid (0.1% v/v) had any effect on the localization of Ire1p-GFP (**Figure 6A**). When cells were simultaneously treated with 0.1% acetic acid and 5% ethanol, the formation of the punctate spots of Ire1p-GFP was clearly and quickly induced. Additionally, the transcription level of *KAR2* was also significantly elevated by the combined stress of 0.1% acetic acid and 5% ethanol (**Figure 6B**). These results clearly indicate that the combined stress caused ER stress and activation of the Ire1p-Hac1p pathway. These results also suggest that ER stress is easily induced in yeast cells during the fermentation process in lignocellulosic hydrolysates.

### Effects of Extracellular pH on the UPR Caused by Acetic Acid

**FIGURE 7:**
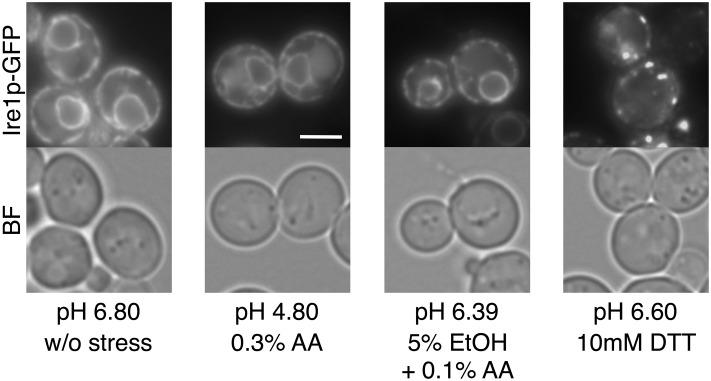
Effects of extracellular dissociated acetic acid on the UPR. Exponentially growing cells were exposed to the indicated conditions for 60 min using the SD-PPB medium whose pH was adjusted to 6.80 by potassium phosphate buffer. Final pH values after addition of acetic acid, ethanol, and DTT into the SD-PPB medium are indicated. Ire1p-GFP was immediately observed after the treatment. Representative pictures are shown. BF, bright field. The white bar indicates 3 μm.

The toxicity of acetic acid is mainly attributed to the undissociated form, due to its ability to enter cells by passive diffusion across the plasma membrane (Guldfeldt and Arneborg, 1998). We next investigated whether extracellular acetic acid in the dissociated form causes ER stress or not. Acetic acid in the dissociated form was obtained by adjusting pH of the medium. We prepared the SD-PPB medium whose pH was adjusted to 6.80 using 100 mM PPB instead of water. The final pH of the SD-PPB medium after addition of 0.3% acetic acid reached to 4.80, which was slightly higher than the p*K*a value of acetic acid (p*K*a = 4.76). As shown in **Figure 7**, 0.3% acetic acid in the SD-PPB medium did not induce changes in the localization of Ire1p, while DTT in the SD-PPB medium induced the formation of punctate spots of Ire1p-GFP regardless of the increased pH of the medium (pH 6.60). A similar invalidation was also obtained for the combined stress of 0.1% acetic acid and 5% ethanol (pH 6.39). These results suggest that the entry of a significant amount of acetic acid into cells is required for activation of the Ire1p-Hac1p pathway. Indeed, the proportion of undissociated form to dissociated form of acetic acid is almost 1:1 at pH 4.8, whereas it is 1:0 at pH ∼3.0 and the intracellular concentration of acetic acid will be much higher.

## Discussion

In the present study, we found that acetic acid causes ER stress and induces the UPR (activation of the Ire1p-Hac1p pathway) in *S. cerevisiae.* A treatment with acetic acid (>0.2% v/v) elevated the levels of misfolded proteins in the ER. Although the effects of acetic acid on processing in the ER have not yet been examined, the present study demonstrated that acetic acid exerted adverse effects on protein folding and processing in the ER and suggests that the accurate synthesis of secretory proteins and transmembrane proteins is disturbed in the presence of acetic acid. In responses to ER stress caused by acetic acid, we clarified that the oligomerization and activation of Ire1 were induced in yeast cells, following the splicing of *HAC1* mRNA and transcriptional activation of *KAR2*. Since *ire1*Δ and *hac1*Δ cells were more sensitive to acetic acid than wild-type cells, activation of the Ire1p-Hac1p pathway appears to play a role in the acquisition of maximum tolerance to acetic acid stress. *AQR1*, *PDR12*, *TPO2*, and *TPO3* encode transmembrane proteins (multi-drug resistance transporters) involved in acetic acid tolerance, and their transcriptions are activated in the yeast adaptive response to acetic acid (Holyoak et al., 1999; Tenreiro et al., 2002; Mira et al., 2010b,a; Tanaka et al., 2012). For the accurate synthesis of these transmembrane proteins, maintaining the capability of protein folding and processing in the ER is required under acetic acid stress. It is presumable that induction of the UPR also contributes to acquire the sufficient tolerance to acetic acid through the recovery and maintenance of the folding ability of proteins in the ER.

According to the types of biomass and pretreatment methods, levels of acetic acid often reach very high in lignocellulosic hydrolysates (Klinke et al., 2004; Nakata et al., 2006; Almeida et al., 2007). Additionally, contamination with lactic acid bacteria and acetic acid bacteria increases the concentration of acetic acid in open fermenters (Beckner et al., 2011). These imply that yeast cells are often exposed to severe acetic acid stress from the beginning to the end of the process of bioethanol production, and there is a high possibility that ER stress persists in yeast cells during the fermentation process of lignocellulosic hydrolysates. Since we only analyzed the yeast response after relatively short-time exposures to acetic acid (15–60 min) in this study, it remains unknown whether yeast cells maintain the activation of the Ire1p-Hac1p pathway or not after a prolonged exposure to acetic acid stress. In mammalian cells, it was reported that Ire1p is deactivated over time even if ER stress remains unmitigated (Lin et al., 2007; Li et al., 2010). We are preparing further research to clarify whether yeast Ire1p is also deactivated or remains to be activated over time during the prolonged exposure to acetic acid stress or during the fermentation process in lignocellulosic hydrolysates. We also detected the synergistic effects of the combination of acetic acid and ethanol. Although neither mild ethanol stress nor mild acetic acid stress individually affected Ire1p localization, their combination clearly induced the activation of Ire1p-Hac1p pathway. This result implies that, even if acetic acid concentration is not high, fermenting lignocellulosic hydrolysates are at a higher risk of the induction of ER stress with increases in ethanol concentrations.

Our results also indicate the diversity of the effects of carboxylic acids on yeast UPR. Acetic acid, propionic acid, and sorbic acid induced the oligomerization of Ire1p-GFP and transcriptional activation of *KAR2*, suggesting their abilities to cause ER stress and activate the Ire1p-Hac1p pathway. On the hand, Ire1p-GFP did not change its intracellular localization by the treatment with lactic acid. The activation of Ire1p was not induced by 2.5–3.0% lactic acid (2.5% lactic acid, pH 2.20; 3.0% lactic acid, pH 2.10), however, it reduced the pH of SD medium at greater than 0.3% acetic acid (pH 3.30). These results suggest that the high acidity of the extracellular environment is not the primary cause of ER stress, and that acetic acid, unlike lactic acid at least for the concentrations tested, exerts its induction effects on ER stress inside yeast cells. Nevertheless, it still appears to be crucial to adjust the pH of the medium in order to prevent ER stress. Dissociated acetic acid hardly enters cells (Guldfeldt and Arneborg, 1998), and medium containing PPB invalidated acetic acid via reducing levels of the undissociated form in the induction of ER stress and the UPR (**Figure 7**). These results suggest the usefulness of adjusting the pH of lignocellulosic hydrolysates in order to prevent ER stress caused by acetic acid and by the combined stress of low concentrations of acetic acid and ethanol. Although the elicitation of ER stress during the process of bioethanol production has not been considered in detail, the results of the present study confirmed that acetic acid and the combination of low concentrations of acetic acid and ethanol cause ER stress and induce the UPR in yeast cells. Our results suggest that maintaining or enhancing the folding ability of proteins in the ER contributes to improving the stress tolerance of yeast and breeding of robust yeast strains for bioethanol production using the lignocellulosic biomass.

## Author Contributions

NK did most of experiments and YK did several important experiments. SI did several experiments and mainly prepared the manuscript.

## Conflict of Interest Statement

The authors declare that the research was conducted in the absence of any commercial or financial relationships that could be construed as a potential conflict of interest.

## Footnotes

1 http://imagej.nih.gov/ij/
